# Treatment of Severe Ocular Mpox with Cidofovir and Tecovirimat

**DOI:** 10.3201/eid3204.250882

**Published:** 2026-04

**Authors:** Xavier Brousse, Rayan Kreidie, Eugénie Mourgues, Luna Fraysse, Marin Lahouati, Vincent Servant, Sonia Burrel, Valentine Saunier, Charles Cazanave

**Affiliations:** Bordeaux University Hospital, Bordeaux, France (X. Brousse, R. Kreidie, E. Mourgues, L. Fraysse, M. Lahouati, V. Servant, S. Burrel, V. Saunier, C. Cazanave); Bordeaux University, Inserm, UMR1034, Pessac, France (M. Lahouati); CNRS UMR 5234, Bordeaux University, Bordeaux (S. Burrel, C. Cazanave)

**Keywords:** Monkeypox virus, mpox, viruses, sexually transmitted infections, keratitis, cidofovir, MSM, France

## Abstract

Mpox, a reemerging zoonotic disease since 2022, primarily affects the skin; ocular involvement is rarely reported. We present a case of mpox-caused disciform keratitis treated with a combination of cidofovir and tecovirimat. The patient recovered without residual ocular sequelae, suggesting these drugs are an option to treat ocular mpox manifestations.

Mpox is a reemerging zoonotic disease, with >140,000 cases reported worldwide since 2022 (*1*). A major outbreak of mpox caused by clade IIb monkeypox virus (MPXV) occurred in Western Europe and the United States in 2022, although MPXV clade I has caused high mortality rates in Central Africa for decades (*2*). MPXV, a member of the Poxviridae family (*3*), is primarily transmitted through close physical contact between humans and typically causes disseminated cutaneomucosal lesions (*4*). Ocular involvement occurs in ≈1%–10% of cases, with a higher frequency in MPXV clade I infections (*5*). Although conjunctivitis is the most common ocular manifestation, more severe manifestations such as keratitis and corneal ulceration have been reported (*6*). All that time data regarding the use of tecovirimat for mpox treatment were limited. Case reports have suggested tecovirimat could have a role in viral clearance, enabling the resolution of lesions (*7*). In severe infections, particularly those with disseminated disease or ocular complications, cidofovir has been used (*8*,*9*). We describe the use of a combination therapy involving tecovirimat and cidofovir for treating a case of mpox-associated disciform keratitis. 

## The Case

A 31-year-old man with an unremarkable medical history was admitted to the ophthalmology clinic with sudden-onset visual acuity loss for the past 72 hours, ocular redness, and pain in the left eye. He was not taking any regular medications, except for on-demand preexposure prophylaxis for HIV. The patient had not received an mpox vaccination and reported recent sexual encounters with men involving unprotected oral sex.

The patient’s recent medical history included a confirmed MPXV infection beginning a month earlier, characterized by disseminated cutaneous lesions and anal ulcerations, later progressing to an anal abscess requiring surgical intervention. MPXV infection was confirmed by PCR from an anal swab. Test results for other sexually transmitted infections, including HIV, were negative.

One week after the anal surgery, at the initial consultation (day 0 [D0]) a slit-lamp examination revealed disciform keratitis characterized by corneal edema, a pseudodendritic ulcer, and conjunctival hyperemia (Figure, panel A). Corrected distance visual acuity (CDAV) was 0.4 logMAR (base 10 logarithm of the minimum angle of resolution). We initiated empirical treatment with oral valaciclovir (1 g 2×/d), followed 48 hours later by topical dexamethasone (3 drops 4×/d, decreasing by 2 drops every 3 days) and neomycin (1 drop 4×/d), on the basis of a presumptive diagnosis of herpes simplex virus (HSV) or varicella-zoster virus (VZV) infection. However, conjunctival swab testing was negative for both HSV and VZV.

Despite initial antiviral drug therapy, follow-up examination on D4 revealed persistent keratitis (Figure, panel B) with no notable clinical improvement (CDAV was 0.7 logMAR). New corneal infiltrates had developed, along with signs of anterior chamber inflammation, including a positive Tyndall effect and central granulomatous retrodescemetic precipitates. Because of the suspicion of a mpox-related ocular complication, we performed an anterior chamber tap on D6. The sample tested negative for MPXV, HSV, and VZV.

We tested a corneal swab on D8 that was positive for MPXV with a cycle threshold (Ct) value of 29. We confirmed the MPXV infection and clade IIb identification by using real-time PCR (*10*,*11*). Retrospective analysis of the initial conjunctival swab from D0 also revealed MPXV positivity, with a Ct value of 29.4. Because of those findings, we discontinued valaciclovir and maintained topical treatment. Despite that approach, the condition progressed to ulcerative keratitis, with visual acuity deteriorating to complete visual blur (counting fingers without CDAV available).

Because of the severity of the ocular involvement and the shortage of other local treatments available at that time in France, and after a multidisciplinary discussion, we initiated second-line antiviral drug therapy consisting of a single intravenous infusion of cidofovir (5 mg/kg) and oral tecovirimat (600 mg 2×/d for 2 weeks). In accordance with clinical guidelines, intravenous cidofovir administration was accompanied by probenecid (1.5 g 3 hours before and 0.5 g 2 hours after cidofovir infusion). We initiated that combination therapy on D15 after the initial ophthalmologic evaluation. Baseline renal function was unremarkable. No proteinuria was detected before treatment. The patient tolerated the antiviral drug regimen well and maintained stable renal function. Topical corticosteroid therapy was continued.

At the follow-up visit on D21, one week after the cidofovir infusion, we observed marked regression of the ocular lesions (Figure, panel C). CDAV improved to 0.2 logMAR. MPXV PCR testing of the conjunctival swab sample was negative. By D29, two weeks post-infusion, we confirmed substantial clinical improvement (Figure, panel D), including complete resolution of corneal edema, conjunctival hyperemia, and inflammatory signs. Visual acuity was almost normal with a CDAV of 0.1 logMAR. Only minor epithelial opacities remained. By D56, the patient had fully recovered, with only faint epithelial opacities persisting, none staining with fluorescein.

**Figure:**
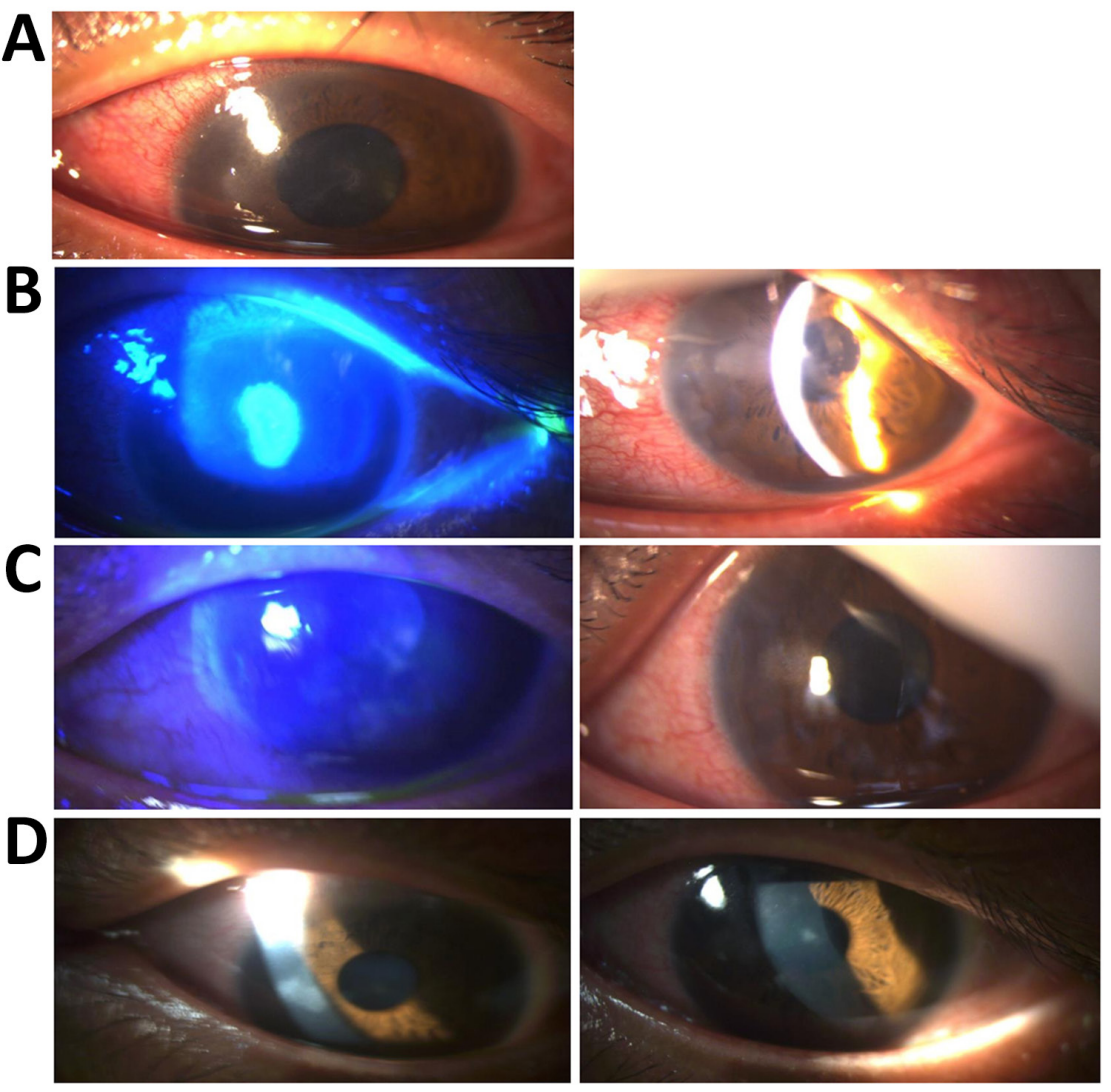
Ophthalmologic evaluation of patient with severe ocular mpox treated with cidofovir and tecovirimat. Images are of patient’s left eye over course of diagnosis and treatment. A) Slit-lamp examination at day 0, showing corneal edema, a pseudodendritic ulcer, and conjunctival hyperemia characteristic of disciform kerititis. B) On day 5, before first-line treatment with valaciclovir, fluorescein staining (left) and slit-lamp examination (right) showing persistent keratitis and formation of new corneal infiltrates. C) On day 21, seven days after initiation of second-line antiviral drug treatment with cidofovir and tecovirimat, fluorescein staining (left) and slit-lamp examination (right) showing marked clinical improvement and reduction in infiltrates. D) On day 29, fourteen days after treatment with second-line antiviral drug treatment with cidofovir and tecovirimat, left and right images are of slit-lamp examination showing complete resolution of corneal edema, conjunctival hyperemia, and inflammatory signs.

## Conclusions

We present the use of cidofovir and tecovirimat to treat a severe mpox infection. Although ocular complications of mpox are rare (*5*), they represent a critical subset of cases with the potential to impair visual outcomes. Those severe manifestations, along with extensively disseminated mucocutaneous lesions, constitute some of the most serious clinical manifestations of mpox. Clinicians should remain alert to the possibility of ocular involvement in patients with mpox, because early recognition and prompt treatment are crucial to preventing complications.

Current data suggest tecovirimat provides limited benefit for infections caused by MPXV clade Ib (*12*). In addition, results from the STOMP trial (*13*), which investigated tecovirimat in patients with MPXV clade IIb infections, revealed no major effect on viral clearance or lesion resolution. The therapeutic options for mpox remain limited, with cidofovir being the primary alternative. Cidofovir has been approved since 1996 for the treatment of cytomegalovirus retinitis in patients with AIDS (*14*). Once phosphorylated, cidofovir inhibits viral DNA synthesis by targeting DNA polymerase (*15*). In contrast, tecovirimat blocks extracellular virus release at a later stage of the viral life cycle, highlighting the potential benefit of combining those agents with distinct mechanisms of action (Appendix Reference 16). The availability of newer antiviral drugs with less nephrotoxicity, such as maribavir (Appendix Reference 17), has led to a limited prescription of cidofovir. However, mpox typically affects younger persons without major underlying conditions, which can reduce cidofovir’s adverse effects.

The treatment of ocular manifestations of mpox remains entirely nonstandardized. The clinical course observed in our case is consistent with previous reports (*9*), supporting the hypothesis that cidofovir might be effectively combined with tecovirimat in this setting. Other promising topical treatments, such as trifluridine, have been proposed for treating this type of infection (Appendix Reference 18). Because of the severity of the ocular involvement, the shortage of this treatment at that time, and the poor visual prognosis, we preferred to use systemic treatment.

Other studies have documented favorable outcomes with tecovirimat monotherapy. Those improvements might reflect the natural course of the disease rather than the specific efficacy of the antiviral drug treatment. For instance, the median time to lesion resolution reported in the literature is ≈29 (interquartile range 25–39) days (Appendix Reference 18), whereas in our case, we observed major clinical improvement within 7 days of initiating treatment, with complete resolution within 14 days. 

Although rare, clinicians should remain vigilant for ocular complications associated with mpox. Cidofovir might hold promise as a therapeutic option in this context, particularly considering its manageable toxicity profile. However, more robust prospective data are needed to confirm those observations. Ocular involvement in mpox warrants prompt therapeutic intervention because of the risk for major and potentially irreversible visual sequelae. Cidofovir, alone or in combination with tecovirimat, might be a therapeutic option to treat ocular manifestations of mpox.

Appendix Additional information about treatment of severe ocular mpox with cidofovir and tecovirimat
