# Supplementary material for: Treatment of Severe Ocular Mpox with Cidofovir and Tecovirimat
Source: Emerg Infect Dis. 2026 Apr;32(4):623–6. doi: 10.3201/eid3204.250882 (PMC13094844; doi:10.3201/eid3204.250882)
Supplement: Appendix — Additional information about treatment of severe ocular mpox with cidofovir and tecovirimat [file 25-0882-Techapp-s1.pdf]

Article DOI: https://doi.org/10.3201/eid3204.250882
EID cannot ensure accessibility for supplementary materials supplied by authors. Readers who have difficulty accessing supplementary content should contact the authors for assistance.

Treatment of Severe Ocular Mpox with Cidofovir and Tecovirimat

Appendix

Appendix References

1. Quenelle DC, Prichard MN, Keith KA, Hruby DE, Jordan R, Painter GR, et al. Synergistic efficacy of the combination of ST-246 with CMX001 against orthopoxviruses. Antimicrob Agents Chemother. 2007;51:4118–24. PubMed https://doi.org/10.1128/AAC.00762-07

2. Avery RK, Alain S, Alexander BD, Blumberg EA, Chemaly RF, Cordonnier C, et al.; SOLSTICE Trial Investigators. Maribavir for refractory cytomegalovirus infections with or without resistance post-transplant: results from a phase 3 randomized clinical trial. Clin Infect Dis. 2022;75:690–701. PubMed https://doi.org/10.1093/cid/ciab988

3. Perzia B, Theotoka D, Li K, Moss E, Matesva M, Gill M, et al. Treatment of ocular-involving monkeypox virus with topical trifluridine and oral tecovirimat in the 2022 monkeypox virus outbreak. Am J Ophthalmol Case Rep. 2023;29:101779. PubMed https://doi.org/10.1016/j.ajoc.2022.101779
